# Hydrocephalus After Gamma Knife Radiosurgery for Vestibular Schwannoma: Favorable Outcomes After Shunt Placement

**DOI:** 10.7759/cureus.25415

**Published:** 2022-05-27

**Authors:** Maxim Marshalik, Kimberly DiManna, Jeffrey Wagner

**Affiliations:** 1 Neurology, HCA Healthcare, Denver, USA; 2 Neurology, Blue Sky Neurology, Aurora, USA

**Keywords:** normal pressure hydrocephalus, timed walk test, shunt, csf tap test, gait assessment, cognitive function, acoustic neuroma, gamma knife radiosurgery, vestibular schwannoma

## Abstract

Recent literature has described the development of a normal pressure hydrocephalus after Gamma Knife radiosurgery in patients with vestibular schwannoma. However, there is minimal detail regarding the clinical course and extent of recovery in these patients following shunt placement. This information would help clinicians weigh the risks and benefits of shunt surgery. We describe the clinical course of two such patients who received shunt placement and made a significant recovery not only in gait but also in their cognitive function. Unlike idiopathic normal pressure hydrocephalus, where patients can have a limited recovery after shunt placement, patients with this form of secondary hydrocephalus appear to make a significant recovery following shunting. Due to the complexity of these patients, it is possible for clinicians to attribute normal pressure hydrocephalus symptoms to neurodegenerative disease or vestibular dysfunction. Thus, it is vital that clinicians have a high index of suspicion for hydrocephalus in vestibular schwannoma patients receiving Gamma Knife radiosurgery so that these patients can be treated early with shunt placement.

## Introduction

Hydrocephalus is a known complication of vestibular schwannoma. Given the variable growth rates of vestibular schwannoma, it is difficult to predict which patients will develop hydrocephalus; however, risk factors such as larger tumor size and older age have been described [1]. More recently, studies have found that the period after Gamma Knife radiosurgery is a particularly high-risk time for the development of hydrocephalus. Previous studies have postulated that this is due to tumor necrosis, which causes CSF protein elevation and malabsorption at the level of the arachnoid granulations [2-3]. Indeed, Lee et al. described a transient increase in tumor volume within three to four years after radiosurgery and argued that during this time tumor volume changed rapidly and the possibility of a protein level change in CSF leading to blockage of arachnoid granulations was high. In this case series, hydrocephalus with elevated intracranial pressure (ICP) could develop anywhere from 1.8-37.8 months after radiosurgery. Among the patients requiring VP shunt, they noted increased ICP evidenced by papilledema and ICP of more than 25 in five out of nine patients. Five of eight patients were also found to have elevated protein [4].

In addition to patients developing communicating hydrocephalus, the development of a more insidious normal pressure hydrocephalus (NPH) has also been described. In a large retrospective study of 284 patients with cerebellopontine angle tumors, 39 patients were found to have evidence of hydrocephalus. Of these, 36 patients (92%) had symptoms that were consistent with NPH [5]. Recent case reports have also described the development of NPH after Gamma Knife in vestibular schwannoma [6-7]. It is unclear if patients with an NPH presentation respond as favorably to shunt placement as patients with communicating hydrocephalus. In idiopathic NPH, improvement in gait is often more evident following CSF shunting, while reports of cognitive improvement are variable [8]. Indeed, there is still clinical equipoise regarding whether the benefits outweigh the risks of shunting such patients [9-10], and this has led to the use of cognitive and gait testing before and after a high-volume lumbar puncture to assess whether patients would benefit from shunt placement. Although this testing could predict improved gait after shunt placement, it has not been as helpful at predicting the extent of cognitive recovery. This information is needed to weigh the risks and benefits of shunt surgery. We describe the clinical course of two such patients who received shunt placement and made a significant recovery not only in gait but also in their cognitive function.

## Case presentation

Patient 1

An 81-year-old female with a right-sided vestibular schwannoma presented with a one-month history of progressing gait instability, cognitive decline, lethargy, and new tremor. She had undergone Gamma Knife radiosurgery 20 months prior to hospitalization. Improvement in symptoms after high-volume lumbar puncture (LP) was used to assess whether the patient would benefit from shunt placement. Montreal Cognitive Assessment (MoCA) and timed walk test were performed before and after lumbar puncture. High-volume lumbar puncture was performed with the removal of 40 ml of CSF. Opening pressure was found to be 23 mmHg, and CSF showed severely elevated protein of 316.2. Although her MoCA did not significantly improve after LP, there was a significant improvement in the timed walk test; thus, the patient underwent ventriculoperitoneal (VP) shunt surgery. Fifty-two days after VP shunt placement, she was seen in the clinic for follow-up. Both her cognitive assessment and timed walk test were found to have significantly improved after the procedure (Table 1).

**Table 1:** Cognitive assessment and timed walk test of patients before and after high-volume lumbar puncture and during follow-up after shunt placement. * Timed walked test was performed with assistance of walker with the exception of patient 1 who, at the 50-day follow-up, no longer required an assistive device. A shorter distance was chosen in the case of patient 2 as the patient did not have the endurance to perform a full nine-meter walk. MoCA: Montreal Cognitive Assessment; VP: Ventriculoperitoneal; LP: Lumbar Puncture

	Assessments	Pre-LP	1 hour post-LP	5 hours post-LP	~50 days after VP shunt placement
Patient 1	MoCA Score	12	11	12	24
	Timed 9 Meter Walk (seconds)*	36	21	28	10
Patient 2	MoCA Score	10	11	11	25
	Timed 6.5 Meter Walk (seconds)*	75	47	Unable to complete	13

Patient 2

A 79-year-old female with left-sided vestibular schwannoma presented with a two-week history of progressing gait instability with increased falls, cognitive decline, increased confusion, incontinence of bowel and bladder, and worsening tremor. She had undergone Gamma Knife radiosurgery six months prior to hospitalization. Improvement in symptoms after a high-volume LP was used to assess whether the patient would benefit from shunt placement. MoCA and timed walk tests were performed before and after lumbar puncture. High-volume LP was performed with the removal of 40 ml of CSF. Opening pressure was found to be 25 mmHg, and CSF showed an elevated protein of 66.2. Although her MoCA did not change significantly after LP, there was a significant improvement in gait; thus, the patient underwent VP shunt surgery. Forty-nine days after VP shunt placement, she was seen in the clinic for follow-up. She had no further episodes of incontinence. Both cognitive assessment and timed walk test were found to have significantly improved after the procedure (Table 1).

## Discussion

The time courses and CSF findings in our patients are consistent with previous reports describing hydrocephalus after radiosurgery in patients with vestibular schwannoma. Patient 1 and Patient 2 had elevated CSF protein of 316.2 and 66.2, respectively. This is consistent with prior reports [3] and the hypothesis that protein blockage of arachnoid granulations is responsible for the development of hydrocephalus. The delay between onset of hydrocephalus and Gamma Knife radiosurgery in our patients, 20 and six months, respectively, is similar to prior reports and highlights the long window over which hydrocephalus can present.

Families described more chronic and milder symptoms of unclear duration; however, in the weeks prior to hospitalization, there was a significant decline. The hydrocephalus in our two patients presented clinically as normal pressure hydrocephalus with gait instability, cognitive decline, and urinary incontinence. We believe this is a form of secondary normal pressure hydrocephalus that is very responsive to CSF shunting. Previous studies have found that patients with secondary hydrocephalus from a known etiology tend to do better after shunt placement. A large analysis of available data on secondary NPH outcomes showed improvement in >70% of secondary cases (including >50% “excellent” recovery) [11].

Notable limitations include the following. Given the acute on chronic presentation of these patients and their robust response to CSF shunting, it is also possible that they suffered from a communicating hydrocephalus. Another possibility that we cannot exclude is that these patients, given their age and presentation, would have developed NPH regardless of their vestibular schwannoma and radiosurgery history.

After shunt placement, both patients had a reversal of hydrocephalus symptoms and significant improvement in gait and cognitive function (Table 1). We hope these cases illustrate that hydrocephalus symptoms may be reversible in this group of patients with early shunt placement. Clinicians should monitor vestibular schwannoma patients closely after radiosurgery for signs of hydrocephalus. 

## Conclusions

Hydrocephalus can develop over a long time period in patients with vestibular schwannoma following Gamma Knife radiosurgery. Due to the complexity of these patients, it is possible for clinicians to attribute NPH symptoms to neurodegenerative disease or vestibular dysfunction. The cases in this report emphasize that clinicians evaluating patients with vestibular schwannoma should have a high index of suspicion for hydrocephalus as early shunt placement can reverse their cognitive decline and gait instability.
